# Association between urinary TIMP-2 and IGFBP7 as early biomarkers of AKI and oliguria during liver surgery: a prospective pilot study

**DOI:** 10.1186/cc14371

**Published:** 2015-03-16

**Authors:** F Desmet, M D'Hondt, H Pottel, S Carlier, E Hoste, J Kellum, W De Corte

**Affiliations:** 1 AZ Groeninge, Kortrijk, Belgium; 2 Catholic University Leuven, Kortrijk, Belgium; 3 Ghent University Hospital, Ghent University, Ghent, Belgium; 4 University of Pittsburgh School of Medicine, Pittsburgh, PA, USA

## Introduction

Patients undergoing elective liver surgery have an increased risk for developing AKI [1]. This study was intended to assess [TIMP-2]*[IGFBP7] and its possible association with urine output (UO) in this population. Secondly we sought to compare [TIMP-2]*[IGFBP7] with serum creatinine concentration (Scr).

## Methods

A prospective single-center pilot study performed on 12 patients undergoing elective liver surgery. Serial urine samples were analyzed for [TIMP-2]*[IGFBP7] measured with the Nephrocheck device (Astute Medical, San Diego, CA, USA). Serial Scr was analyzed, UO, blood losses, and mean arterial pressure (MAP) were recorded. Fluid management was standardized, oliguria defined as a UO <0.5 ml/kg/ hour. [TIMP-2]*[IGFBP7] values of >0.3 identify patients at high risk and >2 at highest risk for AKI [2].

## Results

Males comprised 66.7%, median age was 72 years. Median surgical time was 195 minutes. Peroperative median MAP was 71 mmHg (IQR 69; 77). Baseline median GFR was normal in eight patients and decreased in four patients (eGFR >90 and 66.5 ml/ minute/1.73 m^2^ respectively). Median baseline Scr was 0.75 mg/dl (IQR 0.61; 1.10), 0.74 mg/dl (IQR 0.64; 1.04) at ICU admission and 0.74 mg/ dl (0.64; 1.04) on day 1 postoperatively. No difference in Scr and eGFR was seen between these time points (*P *= 0.457 and *P *= 0.517 respectively; repeated-measures ANOVA). Median peroperative and postoperative UO was 0.18 ml/kg/hour (IQR 0.13; 0.23) and 0.93 ml/kg/ hour (IQR 0.79; 1.49) respectively. Median baseline [TIMP-2]*[IGFBP7] was 0.10 (IQR 0.04; 0.34), 2.02 (1.44; 6.23) during surgery, 0.61 (IQR 0.27; 1.22) at ICU admission and 0.74 (0.67; 0.97) on day 1 postoperatively. [TIMP-2]*[IGFBP7] differed at these time points (*P *< 0.0001; repeated-measures ANOVA). Peroperative oliguria was associated with increased [TIMP-2]*[IGFBP7] (*P *= 0.018, chi-squared test).

## Conclusion

This pilot study demonstrated the association between [TIMP-2]*[IGFBP7] increase and oliguria and may therefore indicate kidney damage during liver surgery. As Scr could not differentiate for these changes, patients did not meet the classical biomarker criteria for AKI.
